# A high-impact practice for online students: the use of a first-semester seminar course to promote self-regulation, self-direction, online learning self-efficacy

**DOI:** 10.1186/s40561-021-00151-0

**Published:** 2021-05-07

**Authors:** Jacqueline S. Stephen, Amanda J. Rockinson-Szapkiw

**Affiliations:** 1 College of Professional Advancement, Mercer University, 3001 Mercer University Drive, AACC Building, Suite 310, Office 311, Atlanta, GA 30341 USA; 2 College of Education, The University of Memphis, 3798 Walker Ave.,421C Ball Hall, Memphis, TN 38152 USA

**Keywords:** Self-regulation, Self-direction, Self-efficacy, Online learning, High-impact practice, Undergraduate students

## Abstract

Student enrollment continues to increase in online programs, but there is concern surrounding the reportedly high rates of attrition in online classes compared to face-to-face classes. Undergraduate students are poorly prepared and lack the human agency necessary for success in the online learning environment. To address the lack of persistence of undergraduate online students, universities must create and implement interventions that prepare students for the online learning environment and help them develop as autonomous learners. This study examined whether differences in self-regulation, self-direction, and online learning self-efficacy exist between students participating in an experimental high-impact First-Semester Seminar (FSS) class and a traditional FSS class while controlling for pre-existing factors. A quantitative, quasi-experimental, pretest-posttest research design was used for this study with nonequivalent control groups, and multivariate analysis of covariance (MANCOVA) and follow up analyses of covariances (ANCOVA) were used to analyze the data. MANCOVA results revealed a statistically significant difference between groups. Follow-up ANCOVAs revealed differences between the posttest scores of the traditional FSS class and the high-impact FSS class on the measurements for self-directed learning and self-regulated learning.

## Introduction

Prior to the onset of the COVID-19 pandemic in the United States in March 2020, student enrollment in online programs was already on the rise (Friedman, 2018; National Center for Education Statistics, 2017; Seaman, Allen, & Seaman, 2018), and this growth was projected to continue into 2026 (Hussar & Bailey, 2018). An online program is defined as an academic program in which students complete coursework from a distance through virtual instructional and delivery methods. While online undergraduate enrollment increases, however, high rates of attrition in online classes compared to face-to-face classes are a concern (Bloemer, Swan, Day, & Bogle, 2018; Murphy & Stewart, 2017). Attrition rates in online classes have been documented as 10% to 20% higher than traditional face-to-face classes (Bawa, 2016; Kauffman, 2015), and online persistence rates are low, as well. If students are to continue enrolling in online programs and universities plan to increase their undergraduate online program offerings, then the high rates of attrition in online classes must not be overlooked. Interventions aimed at promoting factors associated with online student persistence are essential to student success and, ultimately, university success as persistence rates are vital to accreditation, funding, and reputation (Tinto, 2017; Yang, Baldwin, & Snelson, 2017).

Persistence in an online class is associated with several factors including self-regulated learning (Barnard, Paton, & Lan, 2008; Lee, Choi, & Kim, 2013), self-directed learning (Brookfield, 2013; Rovai, 2003), and online learning self-efficacy (Prior, Mazanov, Meacheam, Heaslip, & Hanson, 2016; Zimmerman & Kulikowich, 2016). The term *human agency* is used in this study to refer collectively to self-regulation, self-direction, and online learning self-efficacy. While not all factors related to online student persistence are within the institution’s control, human agency can be promoted by the institution to improve persistence rates (Diaz, 2002; Rovai, 2003; Tinto, 1993). If institutions are to promote persistence, they need to help students develop human agency, so they can “ … seek to persist” (Tinto, 2017, p. 254).

To address the persistence of undergraduate online students, universities must proactively create and implement interventions to prepare students for the online learning environment and to help them develop human agency. High-impact practices (HiPs) for residential students have been created to impact success, including persistence, positively. HiPs are practices that involve students as active participants in learning experiences to achieve deep learning, resulting in a positive differential impact (Kuh & O’Donnell, 2013). Studies have shown that initiatives aimed at student success can improve student persistence and retention rates in undergraduate students, whether residential (Hankin, 1996; Kimbark, Peters, & Richardson, 2017; Stupka, 1993) or online (Brewer & Yucedag-Ozcan, 2013). While some universities are starting to develop high-impact practices for online students, the development and research are sparse (Kuep, 2018), and a call for evidenced-based HiPs for online students is needed. These HiPs need to incorporate models of student persistence (Bean & Metzner, 1985; Rovai, 2003; Tinto, 1993) supported by literature on online students (Kuep, 2018; Liu & Adams, 2017; Zimmerman & Kulikowich, 2016).

Therefore, this study examined the influence of a high-impact, First-Semester Seminar (FSS) course on online students’ self-regulation, self-direction, and online learning self-efficacy. The current study examined the impact of an intervention predicated on theories of persistence (Bandura, 1997; Knowles, 1989; Rovai, 2003; Zimmerman, 2002) and research on online undergraduate students’ human agency and persistence (Barnard et al., 2008; Williamson, 2007; Zimmerman & Kulikowich, 2016).

## Conceptual framework

Much of the literature on online student attrition and persistence draws its theoretical framework from research by Tinto, Bean, Metzner, and Rovai, and this study relied on their theories for guidance. Tinto (1987) sought to explain traditional undergraduate student attrition through the Institutional Departure Model, emphasizing factors associated with the institution and the student experience. He later revised his model to include nontraditional learners, focusing on pre-entry attributes of family background, skills, and abilities, prior schooling, student goals and commitment to goals, student experiences at the institution, as well as academic and social integration (1993). Building on the work of Tinto, and Bean (1980, 1982), Bean and Metzner (1985) sought to explain student attrition through the Student Attrition Model, emphasizing factors applicable to nontraditional students, with a focus on academic and psychological variables. Bean and Metzner’s (1985) analysis of attrition factors for nontraditional students culminated in the identification of four variables that influence persistence: (a) academic variables; (b) background and defining variables; (c) environmental variables; and (d) academic and psychological outcomes.

Rovai (2003) synthesized Tinto’s (1993) and Bean and Metzner’s (1985) attrition models in his Composite Persistence Model to address the specific needs of undergraduate students enrolled in online classes. Rovai (2003) incorporated student characteristics (age, ethnicity and gender, intellectual development, academic performance, academic preparation) deemed influential to persistence before admission. Additionally, he incorporated external factors (e.g., finances, hours of employment, family responsibility, outside encouragement, opportunity to transfer, life crises) and internal factors (e.g., study habits, advising, absenteeism, course availability, program fit, current GPA, utility, stress, satisfaction, commitment academic and social integration, goal commitment, institutional commitment, learning community) that can impact student persistence after admission. To address persistence in online students, Rovai (2003) contended that students need specific skills (computer and information literacy, time management, reading and writing skills, and computer-based interaction) before admission to an online class or program. He also argued that online students have specific needs after admission (internal factors of program clarity, self-esteem, identification with the institution, interpersonal relationships, access to services) that help them to persist. Rovai (2003) further maintained that while online students need to be self-directed in their learning, they also “expect a pedagogy that matches their learning style” (p. 11), consequently adding pedagogy (learning preferences and teaching styles) as a necessary internal factor after admission.

**Fig. 1:**
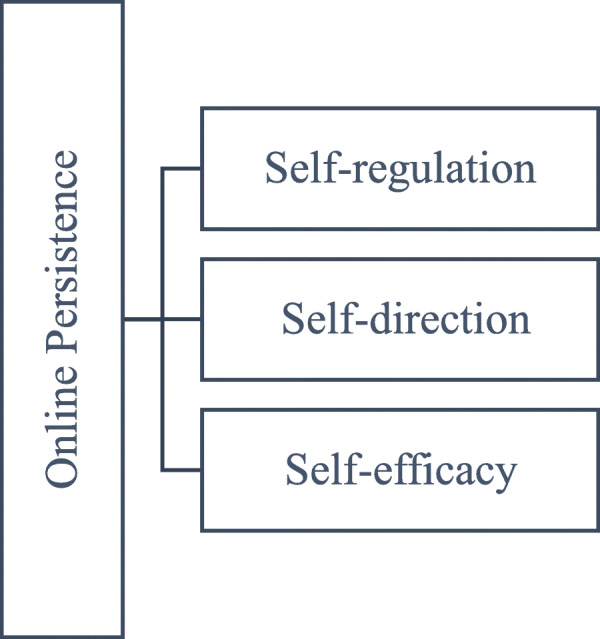
Elements of Human Agency and the Persistence of Online Students

As evidenced by these theories, persistence is complex, and a single intervention cannot address all factors associated with persistence. Therefore, the intervention used in this study was based on what Rovai (2003) identified as internal factors needed to support student persistence in an online class: goal commitment, study habits, and learning preferences. These factors were conceptualized as self-regulation, self-direction, and self-efficacy (Bandura, 1997; Knowles, 1975; Zimmerman, 2002). Undergraduate online students who demonstrate a commitment to their goals, apply effective study habits, and adapt their learning preference are more likely to persist because they are self-regulated (Barnard-Brak, Lan, & Paton, 2010; Knowles, 1975; Zimmerman, 2002) and self-directed (Bandura, 1997; Williamson, 2007) in their learning. Undergraduate online students also need to demonstrate high self-efficacy to persist (Bandura, 1986; Zimmerman, 2000; Zimmerman & Kulikowich, 2016) by committing to their goals, applying effective study habits, and adapting their learning preference. Current research also demonstrates that these three constructs are associated with one another and can be used to predict the persistence of online students (Rockinson-Szapkiw, Holmes, & Stephen, 2019; Stephen, Rockinson-Szapkiw, & Dubay, 2020). Thus, the current study examined the impact of an intervention predicated on theories of persistence and research on online undergraduate students’ human agency and was based on the assumption that the elements of human agency are salient in the persistence of online students (Stephen et al., 2020) and need to be integrated into interventions aimed at improving persistence. See Fig. 1.

## Review of the literature

Not all factors associated with online student persistence are within the institution’s control. Still, research reveals there are factors, such as human agency, within its scope that need to be promoted by the institution to improve persistence rates (Bean & Metzner, 1985; Diaz, 2002; Rovai, 2003; Tinto, 1993). Rovai (2003) emphasized that students must be skilled in time management, computer literacy, information literacy, and computer-based interaction before admission and that they have additional needs (i.e., goal commitment, learning preferences, study habits, interpersonal skills and relationships, self-esteem, accessibility to services) throughout an online class or program influencing their persistence. Yet, undergraduate online students continue to enroll in online classes despite lacking these necessary pre-admission student skills (Broadbent, 2017; Parkes, Stein, & Reading, 2015; You, 2016) and without developing the necessary human agency to persist (Cigdem & Ozturk, 2016; Kizilcec, Pérez-Sanagustín, & Maldonado, 2017; Schommer-Aikins & Easter, 2018; Song, Kalett, & Plass, 2016). Rovai (2003) argued that if institutions are to promote persistence, they need to consider helping students develop human agency, so they can “seek to persist” (Tinto, 2017, p. 254). Hence, institutions assume a key responsibility in helping online undergraduate students develop mechanisms of human agency to persist.

Online student orientation, regular advisement, technology training, and the use of self-assessments to determine student readiness for online learning are some of the strategies that institutions of higher education can employ to support students’ agency, and thus, their persistence (Hart, 2012; Lee & Choi, 2011). For example, one institution’s required orientation centered on the online class environment (i.e., navigation, tool use). An examination of the effectiveness of the orientation found a decrease in online student class withdrawals and an overall increase in student grades (Taylor, Dunn, & Winn, 2015). Another institution of higher education also experienced an increase in online student retention after implementing an online orientation focused on technology use, help-seeking, virtual communication, and tips for success as an online learner (Jones, 2013). While these studies are promising and support the positive impact of such interventions, they were primarily concerned with developing skill and self-efficacy with technology, and the literature surrounding the outcomes and impact of such interventions is sparse (Parkes et al., 2015). Interventions facilitating technology use may enhance technical skills, but students need to develop additional elements of human agency to persist in undergraduate online classes and programs. Those interventions intended to develop human agency need to be examined to determine their impact on online undergraduate student persistence. A study on undergraduate student preparedness for online learning found that students did not feel prepared to navigate an online class, manage their learning, engage with others online, interact with class content, and manage their time (Parkes et al., 2015).

Similarly, Chumbley, Haynes, Hainline, and Sorensen (2018) studied undergraduate online students’ self-regulation. They found that students with limited experience in online learning exhibited anxiety and were unclear on class expectations and their roles and responsibilities. Undergraduate students often fail to persist in online classes and programs because they are unprepared and lack human agency.

Studies have shown that initiatives aimed at student success can improve student persistence and retention rates in undergraduate students, whether residential (Hankin, 1996; Kimbark et al., 2017; Stupka, 1993) or online (Brewer & Yucedag-Ozcan, 2013). Kuh (2008) identified 11 undergraduate residential initiatives regarded as high-impact practices (HIPs) deemed critical to student success and persistence. Kuh described HIPs as experiences that require a considerable investment in time and effort by students; connect learning in the classroom with the real world; encourage collaboration between faculty, students, and other diverse populations; and depend on in-depth feedback. He argued that all higher education institutions should seek to provide at least two HIP experiences for all undergraduate students. Unfortunately, much of the research and focus on HIPs has been aimed primarily at undergraduate, residential experiences. However, online learning continues to grow at an exponential rate, surpassing that of residential higher education programs (Seaman et al., 2018). HIPs, specifically for online environments, have not been identified. Kuh identified 10 HIPs, listed below (Kuh, 2008), and the 11th was added in 2016 (Watson, Kuh, Rhodes, Light, & Chen, 2016). These are:

- First-year experiences (e.g., first year seminars);
- Common intellectual experiences;
- Learning communities;
- Writing-intensive courses;
- Collaborative assignments and projects;
- Undergraduate research;
- Diversity/global learning;
- Service learning, community-based learning;
- Internships; and
- Capstone courses and projects (Kuh, 2008, p. 9–11); and
- ePortfolios (Watson et al., 2016, p. 66).

Salient to this study are First-Year Seminars (FYS), identified by Kuh (2008) as a HIP, given their effectiveness at helping residential undergraduate students to persist (Barefoot, 2004; Tinto, 2012). Researchers have described a FYS as a class designed and structured to assist first-year students in their academic and social development as they transition to learning at the undergraduate college level (Barefoot & Fidler, 1996; Hunter & Linder, 2005).

The content and structure of First-Year Seminars vary across institutions. First-year experience initiatives consist of programs that promote active learning (Eckton & Palfreyman, 2017), study skills (Kimbark et al., 2017), time and stress management (Crisp & Taggart, 2013), relationship-building between students and instructors (Tinto, 2012), awareness of the environment (Tinto, 2012), a sense of belonging and self-efficacy (Tinto, 2012), and institutional expectations (Karp & Bork, 2014). Despite the differences in the characteristics and formats of such interventions, they are critical to student persistence. Tinto (2012) argued that “regardless of the form and focus, evidence of the effectiveness of freshman seminars, when properly implemented, is widespread” (p. 34).

Reflection exercises (i.e., online journals, reflective observations) have been recommended for inclusion in online First-Year Seminars (Kuep (2018) and metanalyses (Panadero, Jonsson, & Botella, 2017) found interventions such as online journals, learning diaries, learning logs, self-assessments, rubrics, scripts, and questionnaires to have positive effects on student self-regulation and online learning self-efficacy. As such, students need to engage in activities designed to help them regularly plan their classwork, monitor their progress, and assess their performance.

### Purpose

The purpose of this quasi-experimental, non-equivalent pretest-posttest control group research study was to examine if differences existed between students participating in an experimental high-impact, First-Semester Seminar (FSS) class and a traditional FSS class on their online self-regulated learning, self-directed learning, and online learning self-efficacy. The independent variable for this study was the type of FSS course the students’ participated. In contrast, the dependent variables and covariates were online self-regulated learning, self-directed learning, and online learning self-efficacy as measured by the Online Self-Regulated Learning Questionnaire (OSLQ) (Barnard-Brak et al., 2010), Self-Rating Scale of Self-Directed Learning (SRSSDL) (Williamson, 2007), and Online Learning Self-Efficacy Scale (OLSES) (Zimmerman & Kulikowich, 2016), respectively. The significance of self-regulated learning, self-directed learning, and online learning self-efficacy to student persistence in online classes commands further examination to determine the design and impact of interventions aimed at supporting online student development of human agency, which the research demonstrates is the combined responsibility of institutions, instructors, and students.

## Methods

### Participant

A convenience sample of undergraduate students was drawn from students who enrolled in a First-Semester Seminar (FSS) course delivered online during the Summer and Fall 2019 semesters at a nonprofit, degree-granting, private institute of higher education in the Southeast region of the United States. The university is categorized by the Carnegie Classification of Institutions of Higher Education as a Doctoral University with High Research Activity (R2). Online undergraduate degree-seeking students enroll in the three-credit, eight-week FSS course during their first semester of study. During the Summer and Fall, 2019 semesters, three were taught as traditional FSS courses, and two courses were taught as experimental High-Impact First-Semester Seminar (FSS) courses. While participants could not be randomly assigned, intact FSS courses were randomly designated as either high-impact or traditional.

Of the 95 students enrolled in the courses, 24 high-impact course members agreed to participate, and 36 traditional FSS course members agreed to participate. Matching was used to control for the selection threat to validity. The 24 high-impact course member volunteers were matched to the traditional FSS course member volunteers based on gender and ethnicity. Age and family data were also considered in the matching process. The decision to create homogenous groups using these factors was based upon research demonstrating that these demographics are often associated and influence self-efficacy, self-direction, and self-regulation (Bidjerano, 2005; Pajares, 2002; Zimmerman & Martinez-Pons, 1990). The use of these variables as covariates was considered; however, the addition of covariates to the analysis would significantly decrease the power of the analysis, especially because the sample size was small.

Therefore, data for a convenience sample of 48 participants were analyzed. Forty-two (88%) of the participants were female, and six (12%) were male. Twenty-four (50%) of the participants reported their ethnicity as Black or African-American, 18 (38%) reported White, and 6 (12%) reported Hispanic or Latino. Students were in different life and family stages. Fifteen (32%) were single with children, 14 (29%) were single with no children, 14 (29%) were married with children, 3 (6%) were married with no children, and 2 (4%) were divorced with children. Thirty-seven (77%) were employed full time, nine (19%) were unemployed, and two (4%) were employed part-time. When asked about previous online learning experiences, 71% (*n* = 34) indicated they had completed an online class in the past, 21% (*n* = 10) indicated they had never taken an online class before, and 8% (*n* = 4) indicated that they had attempted an online class in the past but were not successful.

### Description of the first-semester seminar (FSS)

The FSS class was designed to prepare and orient undergraduate students to college-level online learning. The student outcomes emphasized time management, critical thinking, study habits, study skills, technology use, information literacy skills, knowledge of university academic policies and procedures, access to academic support services and resources, and knowledge of the university culture and history. The eight-week class was delivered synchronously and asynchronously using the university’s learning management system (LMS) and video conference application. The class was structured into four modules, each module spanning 2 weeks and incorporating a variety of activities that supported the three elements of human agency were incorporated throughout the class (Barnard et al., 2008; Bean & Metzner, 1985; Rovai, 2003; Tinto, 1993, 2017). For example, students developed a study and classwork schedule, applied and evaluated the effectiveness of techniques for note-taking, reading, writing, and time management, sought consultation from support systems and resources, set goals, and evaluated their commitment to their goals. Assignments and activities also supported the development of self-direction (Bean & Metzner, 1985; Rovai, 2003; Tinto, 1993; Williamson, 2007). Examples include a student discussion on the competencies of successful online learners, assignments that required students to engage with various support systems across the university, synchronous and asynchronous peer-to-peer learning, completion of a learning preference inventory, and an intelligence self-assessment, and computer and information literacy assignments. Activities also supported online learning self-efficacy (Bandura, 2012; Rovai, 2003; Zimmerman, 2000; Zimmerman & Kulikowich, 2016). For example, students completed a hands-on orientation on the use of the LMS and utilized various synchronous and asynchronous communication tools to seek support from services across the university.

The four sources of self-efficacy were also present in the class. To promote mastery experiences, students were instructed to complete a hands-on self-paced tutorial on the use of the LMS at the start of the class. Vicarious experiences were provided to students through the use of timely and positive feedback from the instructor. Social persuasion was supported through the regular synchronous and asynchronous interactions with individual students and student groups. Physiological factors were addressed through the use of a variety of methods to provide instructions, feedback, encouragement, and support. Finally, recommended practices for online class design and delivery to support students’ self-efficacy (Rovai, 2003; Zimmerman & Kulikowich, 2016) exist throughout the class. Examples include the use of scaffolding for assignments, and the weekly modules are structured to support the learning path.

Despite the incorporation of many recommended practices to foster human agency in the FSS being studied and across interventions in the literature, some students continue to face challenges in managing their time, applying study skills, using appropriate strategies, staying committed to their academic goals, and engaging in self-monitoring and self-evaluation, all of which are instrumental to persistence in an online class (Abdous, 2019; Broadbent, 2017; Heo & Han, 2018; Parkes et al., 2015; Schommer-Aikins & Easter, 2018; You, 2016). As such, the experimental high-impact FSS class incorporated learning logs requiring reflection, which has been identified as a practice that improves human agency (Panadero et al., 2017), and incorporated characteristics of HIPs, including requiring students to invest time and effort, connect learning in the classroom with the real world and apply in-depth feedback. The learning log was only incorporated into the experimental high-impact FSS class. The purpose of introducing the bi-weekly student learning logs was to encourage continuous student engagement and reflection in the learning process. Students’ self-efficacy beliefs influence their decisions to persist by engaging in the learning process (Zimmerman, 1989). Thus, to engage in the learning process through self-management and self-monitoring, self-regulation requires students to develop a sense of self-efficacy, among other factors (Pilling-Cormick & Garrison, 2007). Furthermore, a student’s ability to employ appropriate strategies to engage in the learning process, maintain a commitment to their goals, and manage and monitor their learning has been associated with self-direction (Williamson, 2007). Reflection activities are recommended for inclusion in first-year online seminars (Kuep, 2018), and metanalyses (Panadero et al., 2017) found interventions such as online journals, learning diaries, learning logs, self-assessments, rubrics, scripts, and questionnaires to have positive effects on student self-regulation and online learning self-efficacy.

As each student completed the learning log, the instructor provided feedback within 24 to 48 h in the form of praise, encouragement, suggestions for different strategies (as applicable), and recommended resources and services accordingly (as needed). Effective instructor-student interactions are often a precursor to successful learning experiences (Kuh, Kinzie, Schuh, & Whitt, 2005) and, as Pogue and Ah Yun (2006) noted, instructor immediacy facilitates student learning and affect. Teacher immediacy and presence existed in both classes. Table 1 outlines each learning log prompt, the literature used to inform the development of each question, and the association between each question and the dependent variables in this study.

**Table 1:** Learning log prompts, development of prompts, and their association with self-regulation, self-direction, and online learning self-efficacy

Learning Log Prompt	Modules	Literature Used to Inform Development of Prompts	Associated Dependent Variables
After reviewing the contents of this module, enter 3 to 5 learning goals for this module.	1, 2, 3, 4	• Barnard-Brak et al., 2010; Williamson, 2007; Zimmerman & Kulikowich, 2016	• Self-regulation (goal setting); Self-direction (awareness); Self-efficacy (online learning)
Identify the resources (i.e., Online Writing Lab, Library, Internet, etc.) you will need to accomplish your goals during this module.	1, 2, 3, 4	• Barnard-Brak et al., 2010; Williamson, 2007; Zimmerman & Kulikowich, 2016	• Self-regulation (help-seeking); Self-direction (learning strategies, interpersonal skills); Self-efficacy (online learning)
How many hours during this module, do you plan to dedicate to achieving your goals?	1, 2, 3, 4	• Barnard-Brak et al., 2010; Zimmerman & Kulikowich, 2016	• Self-regulation (time management) • Self-efficacy (time management)
Which days of the week do you plan to work on your goals during this module?	1, 2, 3, 4	• Barnard-Brak et al., 2010; Zimmerman & Kulikowich, 2016	• Self-regulation (time management) • Self-efficacy (time management)
Where do you plan to do your classwork during this module (i.e., Library, home office, dining room, etc.)?	1, 2, 3, 4	• Barnard-Brak et al., 2010	• Self-regulation (environment structuring)
List each graded item due in this module and indicate the grade you hope to achieve on each item.	1, 2, 3, 4	• Barnard-Brak et al., 2010	• Self-regulation (goal setting)
Revisit your goals from the previous module and enter them below. For each goal, indicate whether or not you achieved it. a. If you achieved it, discuss the resources you used to help you achieve the goal, the days/hours you spent on the goal, the location where you completed the work towards the goal, and whether or not you earned the grade you had hoped for. b. If you did not achieve it or earned the grade, you had hoped for, discuss the reasons why you were not able to achieve the goal and what you will do differently in the next module to help you achieve your goals and earn the desired grades.	2, 3, 4	• Barnard-Brak et al., 2010; Williamson, 2007; Zimmerman & Kulikowich, 2016	• Self-regulation (task strategies, self-evaluation); Self-direction (awareness, evaluation); Self-efficacy (online learning, time management, technology use)

### Procedures

After securing the Internal Review Board (IRB), an announcement about the study and invitation to participate was posted in FSS courses offered in Summer and Fall 2019. The announcement included a link to the Informed Consent and pretest survey, which was available 1 week before the start of the class. The pretest survey remained open during the first week of the course. During the final week of the course, individuals who completed the pretest were asked to complete the posttest via another class announcement. Students were asked to provide their university-issued identification number to match the pretest-posttest data.

### Instrumentation

Data for the study were gathered using an online pre and post-survey that included the Online Self-Regulated Learning Questionnaire (OSLQ), Self-Rating Scale of Self-Directed Learning (SRSSDL), and Online Learning Self-Efficacy Scale (OLSES).

#### Online self-regulated learning questionnaire (OSLQ)

The Online Self-Regulated Learning Questionnaire (OSLQ) (Barnard-Brak et al., 2010) was used to measure undergraduate online student self-regulation. It includes the subscales of goal setting, time management, help-seeking, task strategies, and self-evaluation. However, the composite score, including all the subscales, was used for this study. This instrument is comprised of 24 items, each measured on a five-point Likert-type scale (e.g., strongly disagree, somewhat disagree, neither agree nor disagree, somewhat agree, and strongly agree) and have values ranging from strongly agree (5) to strongly disagree (1). The average of all subscales provides a measure of overall self-regulated learning, with higher scores indicating higher levels of self-regulation. The items are presented in the instrument as statements, such as, “I set standards for my assignments in online courses,” “I choose the location where I study to avoid too much distraction,” and, “I prepare my questions before joining in the chat room and discussion.”

#### Self-rating scale of self-directed learning (SRSSDL)

The Self-Rating Scale of Self-Directed Learning (SRSSDL) (Williamson, 2007) was also incorporated into the pretest-posttest to measure undergraduate online student self-direction. Items in the SRSSDL instrument emphasize the areas of awareness (understanding of the factors that contribute to self-directed learning), learning strategies (use of strategies recommended for self-directed learning), learning activities (engaging in self-directed learning activities), evaluation (attributes necessary for self-monitoring), and interpersonal skills (prerequisite skills to becoming a self-directed learner). Combined with the items in the Online Self-Regulated Learning Questionnaire (OSLQ), it yielded additional insight into student skills, strategies, and behaviors that promote persistence. This instrument is comprised of 60 items, equally divided into five categories: awareness, learning strategies, learning activities, evaluation, and interpersonal skills. A Likert-type five-point scale is used for the self-rating of items. The lowest score of one indicates never, a two indicates seldom, a three indicates sometimes, a four indicates often, and the highest score of five indicates always. Higher scores indicate higher self-directed learning behaviors.

#### Online learning self-efficacy scale (OLSES)

The Online Learning Self-Efficacy Scale (OLSES) (Zimmerman & Kulikowich, 2016) was incorporated into the pretest-posttest to measure undergraduate online student self-efficacy in online learning, time management, and use of technology. Combined with the items from the OSLQ and the SRSSDL, it yielded additional insight into student skills and behaviors related to learning in the online environment, time management, and the use of technology for academic purposes. The constructs of interest in this instrument have previously been associated with student success and persistence (Bandura, 2001; Concannon, Serota, Fitzpatrick, & Brown, 2019; Rovai, 2003). While only the composite score was used in this study, this instrument has three subscales, including learning in the online learning environment, time management, and technology use. It is comprised of 22 items with a corresponding six-point scale for each item. The items are presented in the instrument as statements, such as, “Navigate online course materials efficiently,” “Complete all assignments on time,” and “Learn without being in the same room as the instructor.” Students use the six-point scale to indicate their perceptions of their performance on each of the items.

Reliability analyses were calculated for each scale. All three scales demonstrated excellent reliability with a Cronbach’s alpha coefficient of .96 for the OLSES, .95 for the SRSSDL, and the OSLQ had a Cronbach’s alpha coefficient of .90. Reliability for the pretest-posttest measure, as a whole, demonstrated excellent reliability with a Cronbach’s alpha coefficient of .97.

### Data analysis

A multivariate analysis of covariance (MANCOVA) was employed to examine if significant differences on a combination of associated variables of online self-regulated learning, self-directed learning, and online self-efficacy, differed between the traditional FSS courses and the experimental High-Impact FSS while controlling for the covariate (Harlow, 2014; Warner, 2012). The MANOVA was followed by separate analyses of covariance (ANCOVAs) for each dependent variable. The effect size was calculated using the partial eta squared statistic, and interpretation was based on Cohen’s (1977) thresholds of .01for a small effect, .06 for a moderate effect, and .14 for a large effect. The procedures used for each analysis are described in the Results section below.

## Results

Before analyses, assumption testing was conducted. Before the MANCOVA, Pearson’s *r* data analysis revealed significant associations between each pair of dependent variables. Pearson’s *r* values were below the critical cut-off value of .9 (Tabachnick & Fidell, 2007; see Table 2). Therefore, the assumption of no multicollinearity was satisfied. A scatterplot matrix was used to examine the assumption of linearity, and the assumption was found to be tenable. The homogeneity of regression of slopes assumption was tenable as assessed using one-way MANCOVA modeling. The Shapiro-Wilk test was run to check and assure that the univariate normality assumption was. Each class (i.e., traditional and high-impact) on each dependent variable was normally distributed (*p* > .05). The assumption of extreme outliers was assessed. Inspection of the boxplots was used to reveal univariate outliers in the data with values greater than 1.5 box-lengths from the box and univariate extreme outliers with values greater than three box lengths. No outliers were found The Mahalanobis distance values were checked to test for multivariate outliers and normality, and the maximum value for the distance for all cells did not exceed the maximum allowable critical value of 18.47 (Tabachnick & Fidell, 2007). The assumption of homogeneity of variances and covariances was tested using Box’s M test and was found tenable. There were no assumption violations.

**Table 2:** Correlations between the three dependent variables (*N* = 48)

Dependent Variable	Online Learning Self-Efficacy	Self-Directed Learning	Online Self-Regulated Learning
Online Learning Self-Efficacy	–	.68*	.56*
Self-Directed Learning	.68	–	.64*

*P < 0.01

Results of the MANCOVA were statistically significant, Wilks’ Λ = .768, *F* (3, 41) = 4.126, *p* = .012, *partial η2* =. 232. Power was .81, accounting for 81% accuracy of results. Given the significance of the MANCOVA, the univariate main effects were examined using a series of one-way ANCOVAs (analysis of covariance) for each of the three dependent variables separately. The Bonferroni adjusted alpha level of .016 (.05/3) was used as the cut-off value for determining statistical significance for the ANCOVAs (Tabachnick & Fidell, 2007). Significant differences were found between the groups on self-direction (i.e., SRSSDL) and self-regulation (i.e., OSLQ). Students in the high-impact FSS class had significantly higher levels of self-direction and self-regulation than students in the traditional FSS class (see Table 3). While the high-impact FSS class had higher mean scores on the dependent variable of online learning self-efficacy (i.e., OLSES), the difference was not significant (see Table 4).

**Table 3:** ANCOVA results (*N* = 48)

Dependent Variable	*F*	*P*	Partial Eta Squared
Online learning self-efficacy (OLSES)	2.73	.106	.060
Self-direction (SRSSDL)	11.39	.002*	.209
Self-regulation (OSLQ)	6.69	.013*	.135

*P ≤ 0.016

**Table 4:**
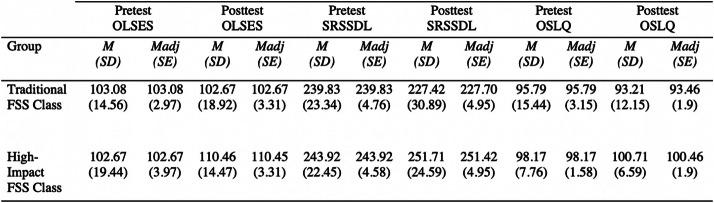
Means, adjustment means, standard deviations, and standard errors for the two groups for each variable

## Discussion

Students in the high-impact FSS class scored significantly higher than students in the traditional FSS class on the Self-Rating Scale of Self-Directed Learning (SRSSDL) and Online Self-Regulated Learning Questionnaire (OSLQ). While the average score on the posttest for the Online Learning Self-Efficacy Scale (OLSES) was higher for the high-impact FSS class (*M* = 110.46) than the traditional class (*M* = 102.67), there was no statistically significant difference between the traditional FSS class and the high-impact FSS class on the dependent variable of online learning self-efficacy. These findings cohere with previous research demonstrating that the inclusion of reflective activities in classes can help foster improvements in students’ time management, application of study skills and strategies, goal commitment, self-monitoring, and self-evaluation (Chang, 2007; Connor-Greene, 2000; Dignath-Van Ewijk, Fabriz, & Büttner, 2015; Zimmerman & Campillo, 2003), all of which have been associated with successful learning and are similar to the constructs of self-regulation and self-direction (Merriam, 2001). The findings can be explained further by theory, which has demonstrated that the three interdependent cognitive processes of self-observation, self-evaluation, and self-reaction are central to these constructs of human agency (Bandura, 1986; Schunk, 2005). Both self-regulation and self-direction require specific cognitive and metacognitive processes. For students to be self-regulated, they need to be aware of their thought process and be motivated to participate actively in the process of meeting the set goal (Zimmerman & Schunk, 2001), which participation in the learning logs required them to do. The incorporation of the required reflection through the learning log assisted students with developing self-regulation and self-direction. Students set goals, identified the resources and strategies they needed to achieve their goals, and evaluated their progress to attribute actions to results. Specific elements of the learning logs may also have contributed to their self-direction. In the learning logs, students were asked to revisit their goals after each module to discuss whether they achieved each goal and elaborate on factors and strategies that helped or hindered their achievement (Knowles, 1975).

While the learning log also emphasized reflection on online learning self-efficacy, there was no statistical difference in the mean scores of the traditional FSS class and the high-impact class in the dependent variable of online learning self-efficacy. Researchers like Zimmerman and Schunk (2011) argue that self-efficacy motivates students to work toward goals and persist in a self-regulated manner. Therefore, it is foreseeable that online learning self-efficacy supported students’ development of self-regulation and self-direction. This idea is supported by the results of Pearson’s *r* correlations analyses in this study, which revealed positive and significant associations between online learning self-efficacy and self-regulation and self-direction.

### Limitations and implications

While there were many significant findings, the study had several limitations, and these limitations provide ideas for future study. This study had limited generalizability due to the small sample size of 48 undergraduate online students from two consecutive semesters, enrolled in a class required by one college of the university. The sample was also unique in that it did not necessarily represent the typical undergraduate population of Caucasian traditional students in the United States (NCES, 2018). Thus, the population for this study is not representative of undergraduate online students at other colleges within the university or undergraduate online students at other universities. Another limitation of this study was the use of the pretest-posttest measure, which was constructed from self-rating instruments that yield results based on a student’s perception of their knowledge, skills, behaviors, and experiences. One of the risks of relying on this self-reported data is the likelihood of participants to overestimate or underestimate their self-regulation, self-direction, and online learning self-efficacy. Students may have rated themselves higher on the pretest-posttest measure because they may have perceived it as a form of assessment, resulting in a ceiling effect. The internal threats to the validity of history and testing may have also been limitations. It may be possible that the differences in pretest-posttest scores were a result of other factors (e.g., activities that occurred in other classes) between the first and second measurements. The study was also limited by non-ignorable, non-response. This study looked only at individuals who completed the pretest and posttest and did not include those who completed the pretest only or chose not to participate at all.

Despite limitations, the findings of this study contribute to the body of knowledge surrounding the use of high-impact practices and interventions to help students develop human agency to persist in online classes and, ultimately, programs. The high rates of attrition in online classes is well-documented in the literature (Bawa, 2016; Jaggars, Edgecombe, & Stacey, 2013; Kauffman, 2015) and must not be overlooked if students are to continue to enroll in online programs and if universities plan to increase their undergraduate online program offerings. As factors that contribute to student persistence in an online class are better understood, universities need to design and develop best practices and interventions aimed at those factors (Tinto, 2017; Yang et al., 2017). Similar to research that identified FSS classes as a high-impact practice for residential students (Barefoot, 2004; Tinto, 2012), the findings of this study support the use of a similar FSS (Barefoot, 2000) as a high-impact practice in the online environment. It is noteworthy that the high-impact FSS class used an intervention emphasizing student self-reflection (i.e., learning logs), which was recommended for inclusion in an online first-year seminar class because of its positive impact on mechanisms of human agency (Kuep, 2018). Results of this study provided evidence that FSS classes for online students need to incorporate reflection activities (i.e., learning logs, self-assessments, rubrics) to help students to develop an awareness of what they did before, during, and after a learning experience.

It is also important to recognize that a key factor of this intervention may have been instructor presence and immediacy. The instructor regularly prompted students to complete the learning log, provided encouragement and feedback, and redirected students to resources and services. Teaching presence, including timely and supportive feedback, is a dimension of the Community of Inquiry (CoI) framework, and research has shown it facilitates student learning in online environments (Garrison, 2017). This implies that as high-impact practices continue to be developed and examined for the online environment that distance education theory and research must be considered in the design.

## Conclusion

While the mechanisms of human agency have been increasingly found as essential to online learning, they have not been well incorporated, especially collectively, into theoretical models that seek to explain online persistence or used collectively to develop interventions in the online environment. Moreover, research that established High-Impact Practices for the online environment are limited. Therefore, this study addressed the gap in the literature by accounting for the three mechanisms of human agency collectively to develop an intervention to influence student success and ultimately persistence, and to provide evidence to an online High-Impact Practice. While semester-to-semester enrollment was not influenced by the intervention, the intervention did influence student self-regulation and self-direction, in which further study needs to examine influence on degree completion. The study findings do however provide evidence for online high-impact practices to improve students’ human agency, and thus, potentially their success.

Finally, as moderate to strong positive associations were found to exist between each mechanism of human agency (i.e., self-efficacy, self-directed learning, and self-regulated learning) and two of the constructs were found to be significantly affected by the High-Impact First-Semester Seminar (FSS) class, this study supports Schunk and Zimmerman’s (1997) assertation that human agency mechanisms, such as self-regulation, are learned and influenced socially, supporting application of theory to high-impact practices in online environments.

## Data Availability

The datasets used and/or analysed during the current study are available from the corresponding author on reasonable request.

## Abbreviations

ANCOVA: Analyses of Covariances
COVID-19: Coronavirus disease of 2019 (Center for Disease Control)
FSS: First-Semester Seminar
FYS: First-Year Seminar
GPA: Grade Point Average
HiP: High-impact practice
IRB: Internal Review Board
LMS: Learning management system
MANCOVA: Multivariate analysis of covariance
NCES: National Center for Education Statistics
OLSES: Online learning self-efficacy scale
OSLQ: Online Self-Regulated Learning Questionnaire
R2: Doctoral Universities – High Research Activity
SRSSDL: Self-Rating Scale of Self-Directed Learning
